# Structure of a hinge joint with textured sliding surfaces in terrestrial isopods (Crustacea: Isopoda: Oniscidea)

**DOI:** 10.1186/s40851-021-00177-9

**Published:** 2021-05-11

**Authors:** Miloš Vittori

**Affiliations:** Department of Biology, Biotechnical Faculty, University of Ljubljana, Večna pot 111, 1000 Ljubljana, Slovenia

**Keywords:** Woodlouse, Tribology, Cuticle, Exoskeleton, Crustacean, Ultrastructure

## Abstract

**Background:**

The study of joints in terrestrial arthropods can provide insights into the evolutionary optimization of contacting surfaces that slide without lubrication. This work reports on the structure of the joint between the propodus and the dactylus in terrestrial isopods, the most successful group of crustaceans on land, focusing on the woodlouse *Porcellio scaber*.

**Methods:**

The joints were studied using fluorescence microscopy, 3D reconstruction, scanning electron microscopy and transmission electron microscopy. The obtained results were functionally interpreted using high-speed video recordings by analyzing the use of the joint during locomotion.

**Results:**

In the joint, which allows the dactylus to move in a single plain, a semicircular process on the propodus fits into a groove on the dactylus and guides its movement. The sliding surfaces of the propodal process are textured in the form of parallel epicuticular ridges a few hundred nanometers thick. This texturing is selective: while the less heavily loaded surfaces are textured, the surfaces that support the isopod during standing and walking are smooth. In contrast, the groove on the dactylus is completely smooth. We found a similar surface texture in several other species of terrestrial isopods and one aquatic isopod.

**Conclusions:**

The selective texturing of the joint may reduce wear by eliminating small particles. This effect of the ridges was confirmed using electron microscopy. The absence of ridges on heavily loaded surfaces may enhance the dissipation of forces in these regions.

**Supplementary Information:**

The online version contains supplementary material available at 10.1186/s40851-021-00177-9.

## Background

Arthropods derive their name from one of their essential features: their jointed appendages. The joints in these appendages allow the rotation of hard skeletal elements, the podomeres, which are connected by flexible arthrodial membranes that enable their movement. Because the cuticle is the outermost layer of the arthropod integument, these joints have contact surfaces on the outside of the body, unlike vertebrate joints. This holds great potential for biomimetics, as the structure of such joints can be mimicked in machine elements, while understanding the mechanisms of friction and wear reduction in arthropod joints can deepen our understanding of tribological principles at the microscopic scale.

Terrestrial isopods (Oniscidea) are the most successful group of terrestrial crustaceans, with over 3700 known species inhabiting environments from seashores to deserts [1]. The pereons (thoracic segments 2–8) of terrestrial isopods bear seven pairs of walking appendages, the pereopods, each consisting of six free podomeres [2]. The penultimate and ultimate podomeres of the pereopods are the relatively long propodus and the much shorter dactylus, respectively. The dactylus is the podomere that makes contact with the substrate and bears a single claw in terrestrial isopods [3]. In a previous description of the pereopods in various isopods, it was noted that the joint between the two distal podomeres differs considerably from other joints. Whereas most crustacean joints rotate about one or two condyles, the joint between the propodus and the dactylus in isopods is supported on one side by a flat cuticular plate [2, 4]. The fine structure of this fundamentally different joint has not previously been investigated. This study reports on the architecture of the propodus-dactylus joint in the rough woodlouse (*Porcellio scaber*), with additional observations in four other isopods. It presents the use of the joint during locomotion and discusses how its structural features may be adaptive.

## Materials and methods

### Isopods

A culture of *P. scaber* was maintained at room temperature (21 °C) with a natural day and night rhythm in Ljubljana, Slovenia. The animals were provided with hazel leaf litter, carrots and potatoes as food. Adults, approximately 1 cm in length, that did not exhibit sternal CaCO_3_ deposits indicative of the premolt stage of the molt cycle [5] were killed by decapitation and their pereopods dissected. Specimens of other isopod species were fixed in 96% ethanol upon sampling. Oniscideans were collected individually by hand. Specimens of *Sphaeroma serratum* were collected underwater with a handheld net after disturbing the substrate. The species used and their sampling localities are listed in Table 1.

**Table 1:** Sampling localities of the isopods used in this study

Species	Sampling locality
*Cylisticus convexus* (De Geer, 1778)	Komen, Slovenia (45°48′44″N, 13°44′28″E); garden
*Ligidium hypnorum* (Cuvier, 1792)	Tanča Gora, Slovenia (45°31′43″N, 15°08′51″E); sinkhole in deciduous forest
*Porcellio scaber* Latreille, 1804	Črnomelj, Slovenia (45°34′05″N, 15°11′18″E); garden
*Sphaeroma serratum* (Fabricius, 1787)	Ankaran, Slovenia (45°34′44.9″N, 13°43′50.4″E); gravel beach
*Titanethes albus* (C. Koch, 1841)	Planina Cave, Slovenia (45°49′08″N, 14°14′42″E); limestone cave

### Fluorescence microscopy

For observation by fluorescence microscopy, pereopods were fixed in a mixture of 2.5% glutaraldehyde and 1% paraformaldehyde in 0.1 M HEPES buffer (pH = 7.3) and decalcified for 48 h in 10% aqueous ethylenediaminetetraacetic acid (EDTA). To image the muscles, the pereopods were stained with 0.1% eosin in 70% ethanol containing 0.1% acetic acid for 48 h and mounted in glycerol. To visualize the cuticle, soft tissues were removed by incubation in 20% aqueous potassium hydroxide (KOH) at 60 °C for 4 h, followed by staining of the cuticle in a 0.5% aqueous solution of Congo red for 48 h (modified after [6]). Optical clearing was performed in a mixture of benzyl alcohol and benzyl benzoate [7].

Imaging was performed using an AxioImager Z1 microscope equipped with an AxioCam MRm camera (both from Zeiss). Eosin fluorescence was imaged using Zeiss filter set 09 (blue excitation; 450–490 nm), and Congo red fluorescence was imaged using Zeiss filter set 15 (red excitation; 546 nm). Optical sectioning was performed using the ApoTome system for structured illumination imaging (Zeiss). Spiers software [8] was used for 3D reconstruction from optical sections.

### Scanning electron microscopy (SEM)

For SEM, pereopods were fixed in 96% ethanol, washed with acetone and dried in hexamethyldisilazane (HMDS). In the case of *P. scaber*, additional sets of pereopods were air-dried without fixation or washing. To expose the sliding surfaces of the joint, propodi and dactyli were separated with a pair of entomological pins. Samples were sputter-coated with platinum using an SCD 050 sputter coater. Images were acquired using a JSM-7500F field emission scanning electron microscope. Measurements within SEM images were performed using FIJI software [9].

### Transmission electron microscopy (TEM)

For the preparation of resin sections, pereopods were fixed and decalcified as described for fluorescence microscopy. After postfixation with 1% aqueous osmium tetroxide, they were embedded in Spurr’s resin (Structure Probe, Inc.). Thin sections (70 nm) were cut on an ultramicrotome (Reichert) using a diamond knife (Diatome). Sections were collected on copper grids and contrasted with uranyl acetate and lead citrate. Images were acquired using a Philips CM 100 transmission electron microscope equipped with cameras 792 BioScan and Orius 200 (both from Gatan).

### Analysis of joint use during locomotion

To visualize the movements of the propodus and dactylus during locomotion, walking of *P. scaber* was recorded at 960 fps using a Samsung Galaxy S9 smartphone camera. Video frames were extracted using VLC media player (VideoLan). The outlines of the pereopods and the isopod body were drawn using Illustrator (Adobe).

## Results

### Morphology of the propodus-dactylus joint

**Fig. 1:**
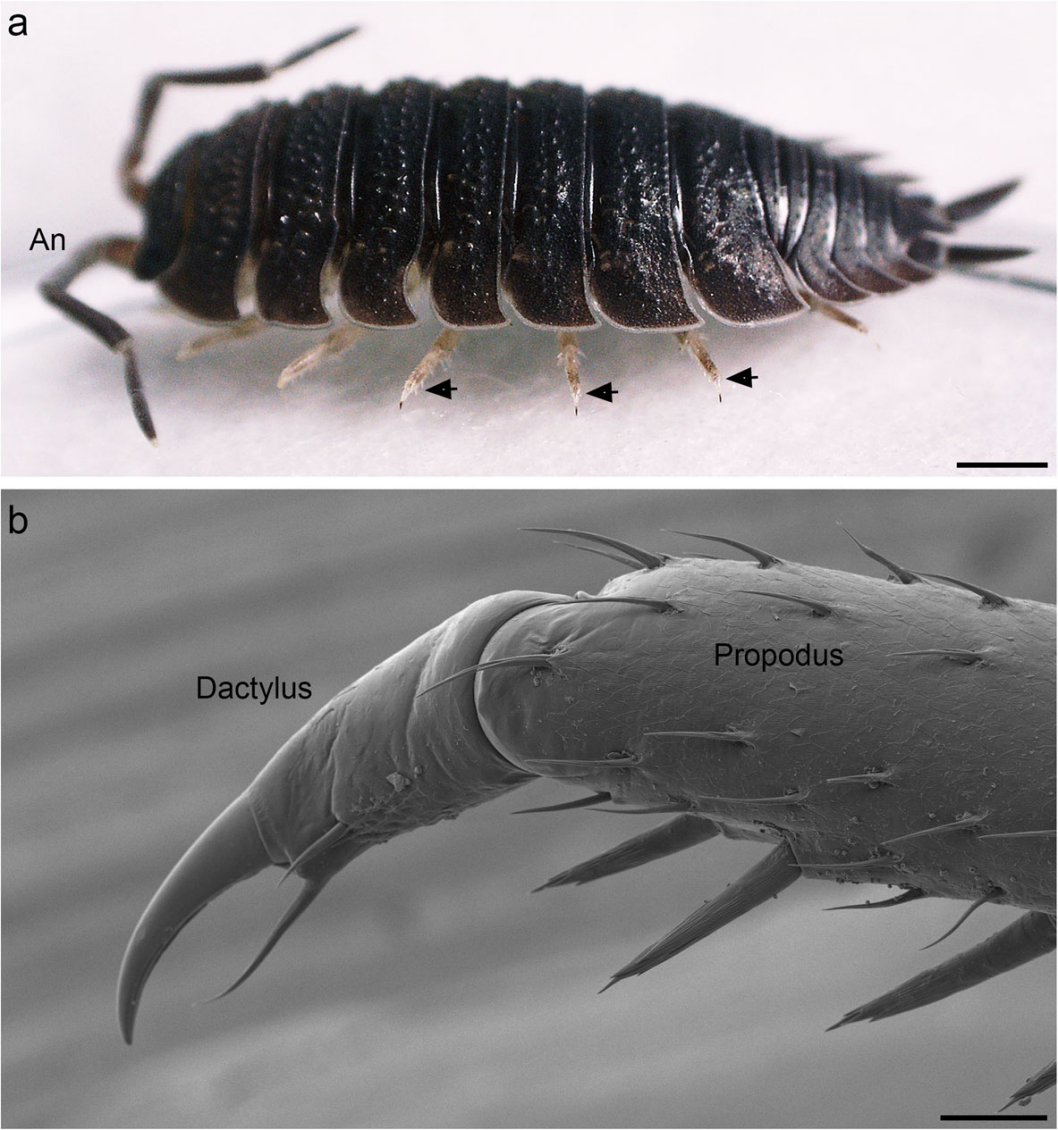
Woodlouse *Porcellio scaber* and its propodus-dactylus joints. **a** Photograph of a specimen of *P. scaber*. The isopod is approximately 10 mm long and stands on the tips of its dactyli. Arrows mark the joints between the propodi and the dactyli of three pereopods. **b** Posterior view of a *P. scaber* pereopod, showing the joint between the propodus and the dactylus. Scale bars: 1 mm (a), 50 μm (b)

**Fig. 2:**
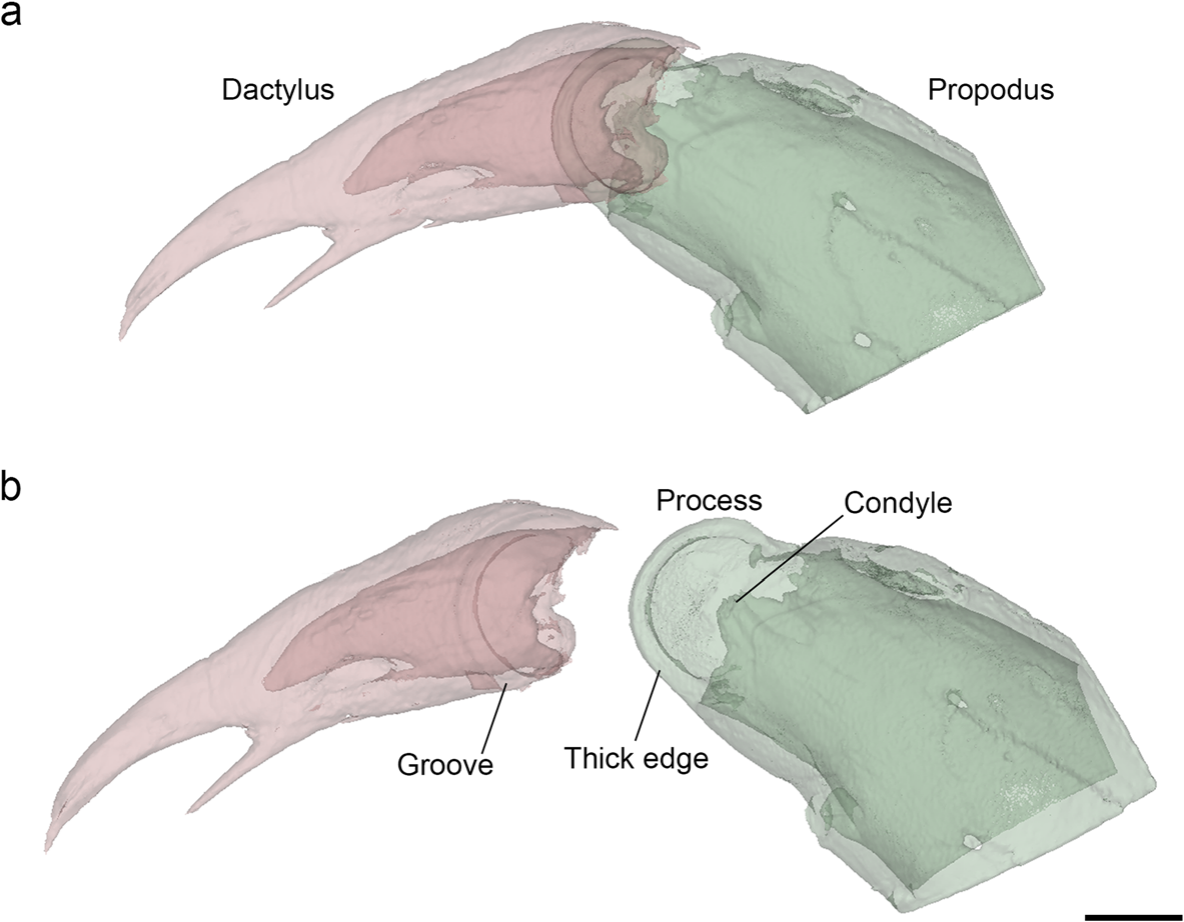
3D reconstruction of the propodus-dactylus joint in *Porcellio scaber.* **a** A propodus-dactylus joint, reconstructed from serial optical sections, posterior view. The propodus is shown in green and the dactylus in red. The arthrodial membrane is not shown. **b** Propodus and dactylus shown separately. The thick edge of the process on the propodus fits in a groove on the dactylus. The propodus also forms a small condyle on the anterior side of the joint. Scale bar: 50 μm

In all seven pairs of pereopods of *P. scaber*, the propodi and dactyli are connected by similar hinge joints (Fig. 1). On the posterior side of the propodus-dactylus joint, the propodus forms a semicircular flat process, the edge of which is thickened and fits into a semicircular groove on the dactylus (Figs. 1 and 2). This process corresponds to the cuticular plate described by Hessler [2]. In adult specimens of *P. scaber*, the propodal process has a radius of 40 μm. Its thickened edge is 7–8 μm wide and projects 10 μm from the surface of the process. As can be shown with optical sectioning, the propodal process is solid—it contains no cavity. The groove on the dactylus is approximately 15 μm deep, and its width and radius correspond to those of the edge of the propodal process that the groove accommodates. This allows both the concave and the convex sides of the process to come into contact with the groove. The dactylus rotates relative to the propodus by sliding along the thick edge of the propodal process, using it as a curved rail. A small condyle is located at the anterior side of the joint (Fig. 2).

### Attachment of muscles that move the dactylus

**Fig. 3:**
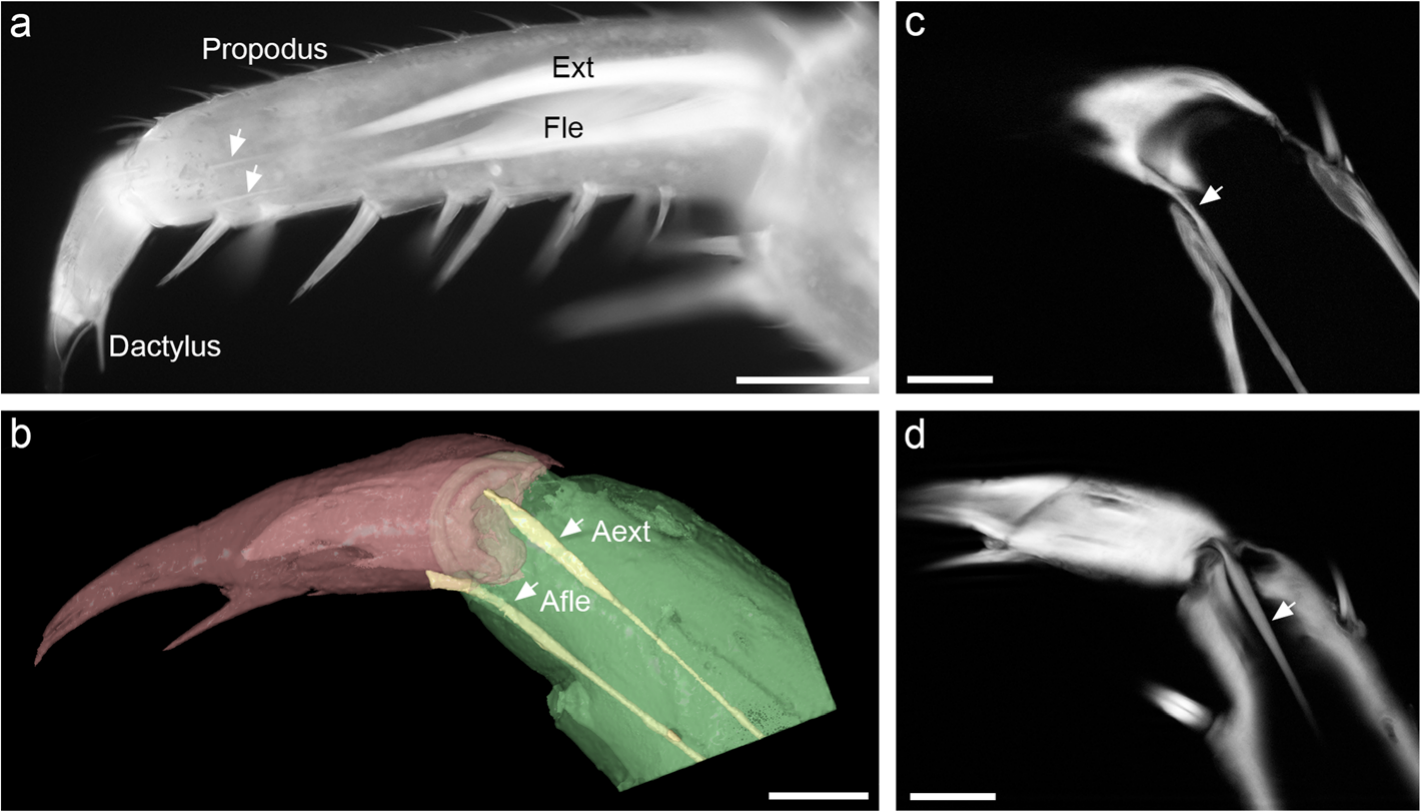
Muscles that move the dactylus. **a** Fluorescence micrograph of a pereopod of *Porcellio scaber*, showing the flexor (Fle) and extensor (Ext) muscles of the dactylus. Each muscle attaches to a long apophysis (arrows). **b** Apophyses (yellow), shown in a 3D reconstruction of the propodus-dactylus joint. The apophysis of the flexor (Afle) attaches ventrally in the medial region and the apophysis of the extensor (Aext) dorsally in the anterior region of the dactylus. **c** Optical section through the medial region of the joint, showing the apophysis of the flexor (arrow). The dorsal margin of the joint is to the upper right of the image. **d** Optical section through the anterior region of the joint, showing the apophysis of the extensor (arrow). Scale bars: 100 μm (a), 50 μm (b, c, d)

Two antagonistic muscles move the dactylus, which is thus capable of rotation in a single plane. The muscles originate proximally in the propodus and insert along a pair of long apophyses, i.e., solid, rod-like extensions of the cuticle. Both apophyses extend from the cuticle at the base of the dactylus, close to the joint (Fig. 3a). The apophysis of the flexor attaches medially on the ventral side, close to the ventral end of the groove on the dactylus (Fig. 3b and c). The apophysis of the extensor attaches dorsally on the dactylus, on the anterior side of its articulation with the propodus (Fig. 3b and d).

### Structuring of the joint surface

**Fig. 4:**
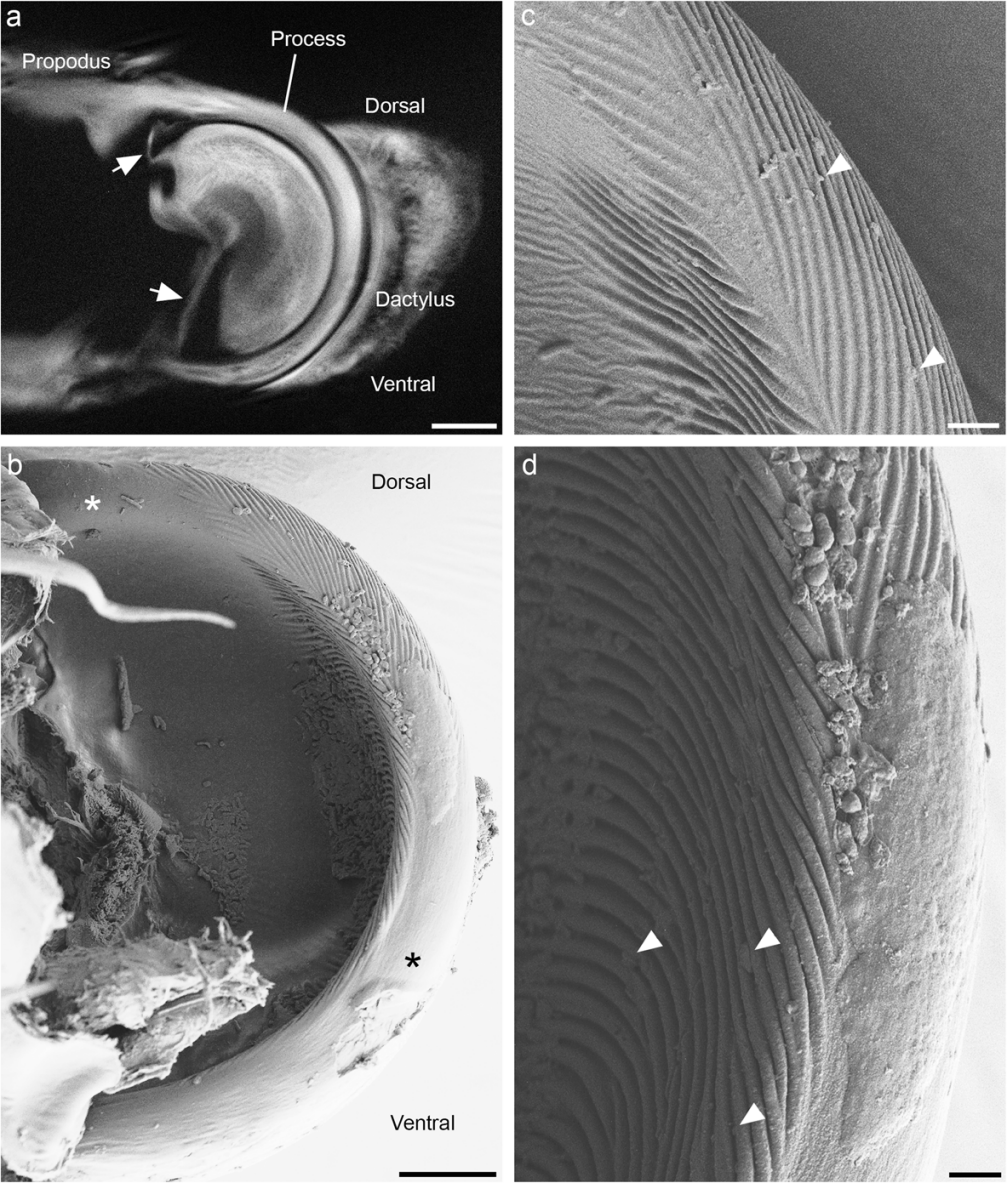
Structure of the propodal process. **a** Optical section through an intact propodus-dactylus joint illustrating how the edge of the propodal process fits into the groove of the dactylus. An arthrodial membrane (arrows) connects the two podomeres. **b** Scanning electron micrograph of the propodal process with the dactylus removed. The tip of the propodus ends with a semicircular cuticular plate with a thickened edge. Parallel ridges are present on the concave surface of the thick edge; on its convex surface, they are only present dorsally. The convex surface of the ventral half of the edge and the concave surface of its most dorsal part are smooth (asterisks). **c** Higher magnification image of the dorsal part of the propodal process, showing the oblique ridges arranged in a herringbone pattern. Arrowheads point to particles lodged between the ridges on its surface. **d** Detail of the ventral part of the edge, showing the ridges on its concave surface and its smooth convex surface. Particles are visible between the ridges (arrowheads). Scale bars: 20 μm (a), 10 μm (b), 2 μm (c and d)

**Fig. 5:**
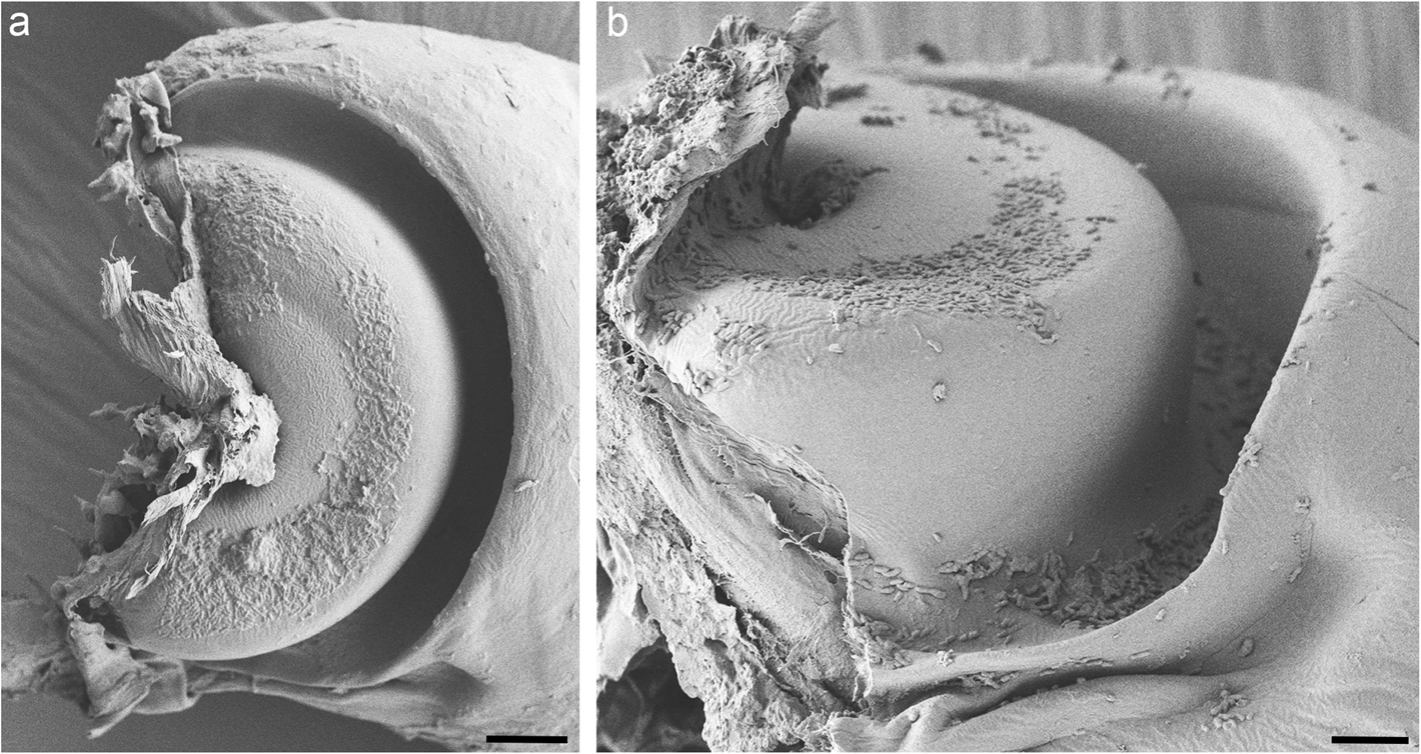
Structure of the groove on the dactylus. **a** Scanning electron micrograph of the base of the dactylus, posterior view. A semicircular groove is visible. **b** The groove on the dactylus, ventral view. The surface of the groove is completely smooth. Scale bars: 10 μm (a), 5 μm (b)

As can be seen with SEM, the surface of the thick edge on the propodal process is textured, with oblique sinusoidal ridges (Fig. 4). This surface texturing is not uniform. On the dorsal segment of the process, the concave surface of the thick edge is smooth, and only its convex (dorsal) surface is textured. On the ventral half of the process, this pattern is reversed: the convex surface of the edge is smooth, and only its concave surface is textured (Fig. 4b). In *P. scaber*, the average spatial frequency of ridges was 610 ± 150 nm (mean ± standard deviation), and their height was 300 ± 40 nm. The ridges are oriented at an angle of approximately 20° relative to the sliding direction of the dactylus. The ridges on the convex surface of the edge mirror the orientation of the ridges on its concave surface, forming a herringbone pattern on the dorsal part of the propodal process (Fig. 4c). Small particles can be observed in the grooves between the ridges on the propodal process (Fig. 4c and d). In contrast to the surface of the propodal process, the entire surface of the groove on the dactylus is smooth (Fig. 5).

The distribution of ridges, which are limited to the propodus, results in contacts between a structured and a smooth surface along most of the thick edge of the propodal process. Contacts between two smooth surfaces occur on the concave side of its dorsal segment and on the convex side of its ventral half. Furthermore, since only the propodal surfaces are structured, the orientation of the dactylus does not influence whether structured or smooth surfaces of the propodal process are loaded when a pereopod supports the body. This depends only on the orientation of the propodus.

### Ultrastructure of the joint cuticle

**Fig. 6:**
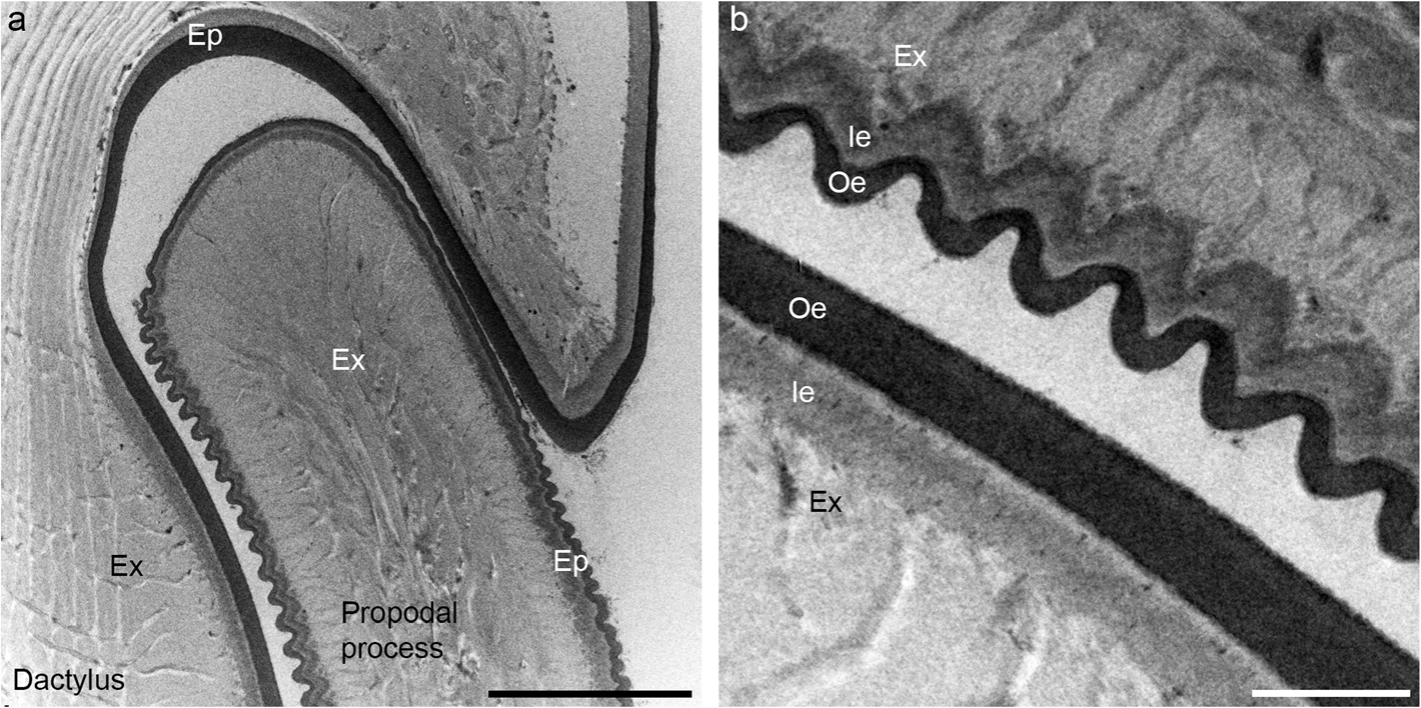
Ultrastructure of the contact surfaces in the propodus-dactylus joint. **a** Transmission electron micrograph of the propodal process inside the groove of the dactylus. The ridges on the propodal process are formed predominantly by the epicuticle (Ep), while its interior consists of the exocuticle (Ex). The epicuticle covering the groove of the dactylus is smooth and thicker. **b** Detail of the sliding surfaces of the propodus (top right) and the dactylus (bottom left). The denser outer epicuticle (Oe) covers the lighter inner epicuticle (Ie). The exocuticle (Ex) is visible in the innermost parts of the ridges on the propodus, while the ridges consist mostly of the epicuticle. The smooth epicuticle of the dactylus is thicker. Scale bars: 5 μm (a), 1 μm (b)

The micrographs obtained with TEM (Fig. 6) confirm the distribution and size of the ridges on the propodal process determined with SEM. The ridges are sinusoidal in cross-section and consist mainly of the epicuticle, which is 600 nm thick in this region (Fig. 6b). The intact epicuticle of the smooth region, which is visible with TEM, demonstrates that the smoothness of the ventral half of the propodal arch is its original structure and not the result of wear. The bulk of the propodal process is formed by the exocuticle, whereas the endocuticle is not visible on cross-sections through it (Fig. 6a). The epicuticle lining the groove of the dactylus has no texturing and is 1 μm thick (Fig. 6). It is therefore much thicker than the epicuticle of tergites, which are covered by a layer of epicuticle approximately 200 nm thick [10, 11].

### Use of the joint

In standing isopods, all 14 pereopods are in contact with the ground and rest on the tips of the dactyli (Fig. 1). During standing, the pereopods are flexed at the joint between the basis and ischium. This causes the basis to point medially and the other podomeres to point laterally. As the propodi are positioned approximately horizontally, the dactyli are flexed and point downward (Fig. 1).

Detailed analyses of the isopod gait were presented by Alexander [12] and Hessler [2]. In this work, high-speed video was used to visualize the positions of the propodus and the dactylus in order to interpret how the joint between them is loaded when it is in use (see Additional file 1).


**Additional file 1** Locomotion of *Porcellio scaber.* High-speed video of a *Porcellio scaber* female during locomotion.

**Fig. 7:**
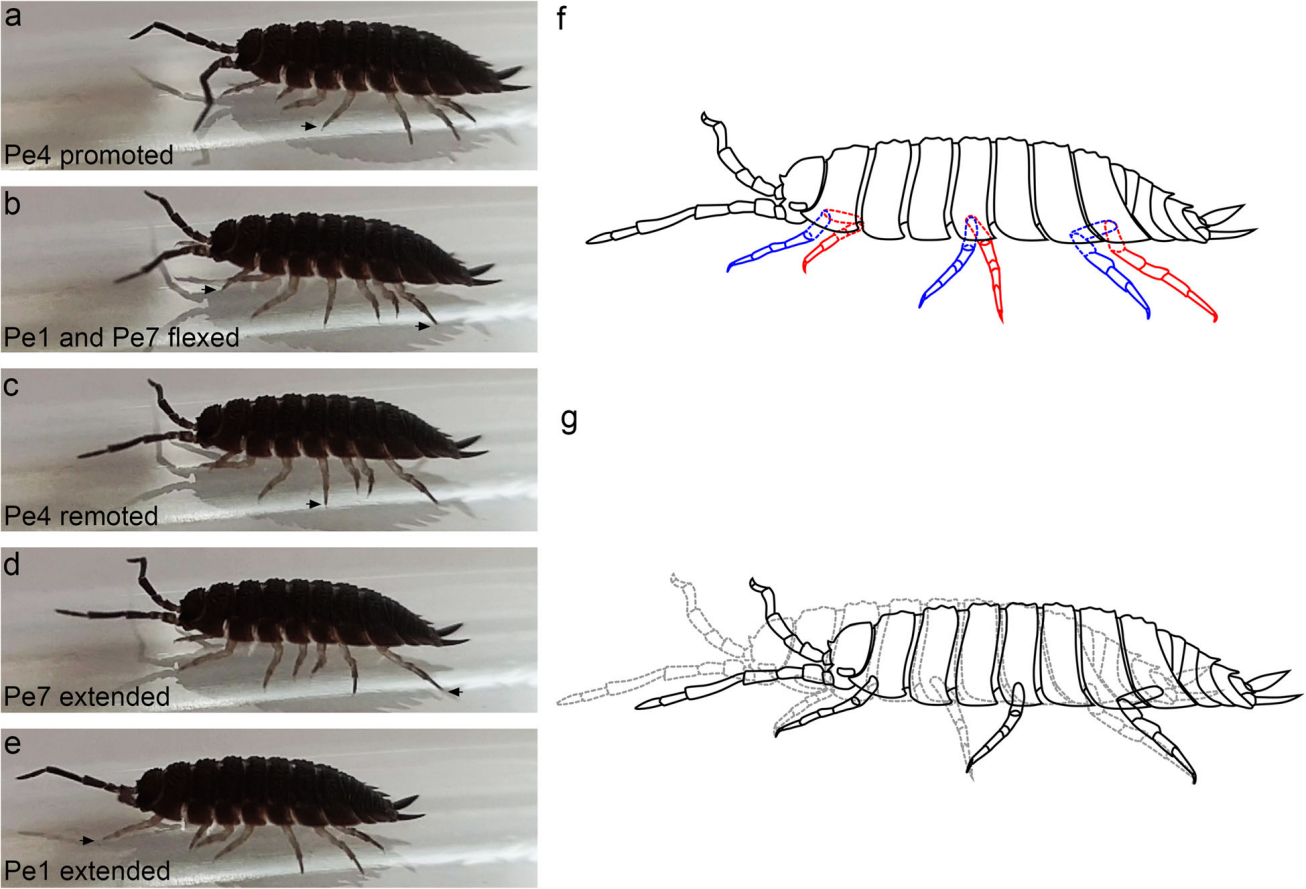
Locomotion of *Porcellio scaber.* **a-e** Images of a walking *P. scaber*, taken at different time points, showing the extreme positions of pereopods 1, 4 and 7 during their effective strokes. Tips of the pereopods are marked with arrows. **f** Illustration of the extreme positions of pereopods 1, 4 and 7 during the beginning (blue) and the end (red) of their effective strokes. Podomeres that are partially concealed by the epimeres are shown with segmented lines. While pereopods 1 and 7 employ flexion and extension of their proximal podomeres, pereopod 4 rotates at its base. **g** Movement of the body relative to the dactyli during the effective strokes of pereopods 1, 4 and 7. The black solid outline shows the starting position, and the gray segmented outline shows the position at the end of an effective stroke. As the pereopods move, they pivot on the dactylus

Most pereopods (pairs 3 to 6) are promoted and remoted by rotation of the basis relative to the coxal plate. During the effective stroke, remotion of the pereopods while pivoting on the dactylus shifts the body forward (Fig. 7). This movement is enabled by the monocondylar ball-and-socket joint between the basis and the coxal plate [2, 13]. The promotion and remotion of the pereopods are associated with very little movement of the other podomeres, which move in a single plain, lifting the pereopods off the substrate and lowering them again during the recovery stroke. During the effective stroke, the propodi of these legs do not change their near-horizontal orientation relative to the substrate, and the dactyli are flexed throughout the effective stroke (Fig. 7).

Pereopods 1, 2 and 7 extend and flex more during locomotion. They move by extending and flexing the basis and ischium relative to the coxal plate, with only a slight promotion and remotion at the joint between the basis and the coxal plate (Fig. 7). The other podomeres extend and flex to a lesser extent, and the propodus is again positioned almost horizontally throughout the effective stroke. Pereopods 1 and 2 are extended during the recovery stroke and retracted during the effective stroke, pulling the body forward. For pereopod 7, this movement is reversed: this pereopod is flexed during the recovery stroke and extended during the effective stroke (Fig. 7).

### Predicted loading of the joint during the active and recovery strokes

**Fig. 8:**
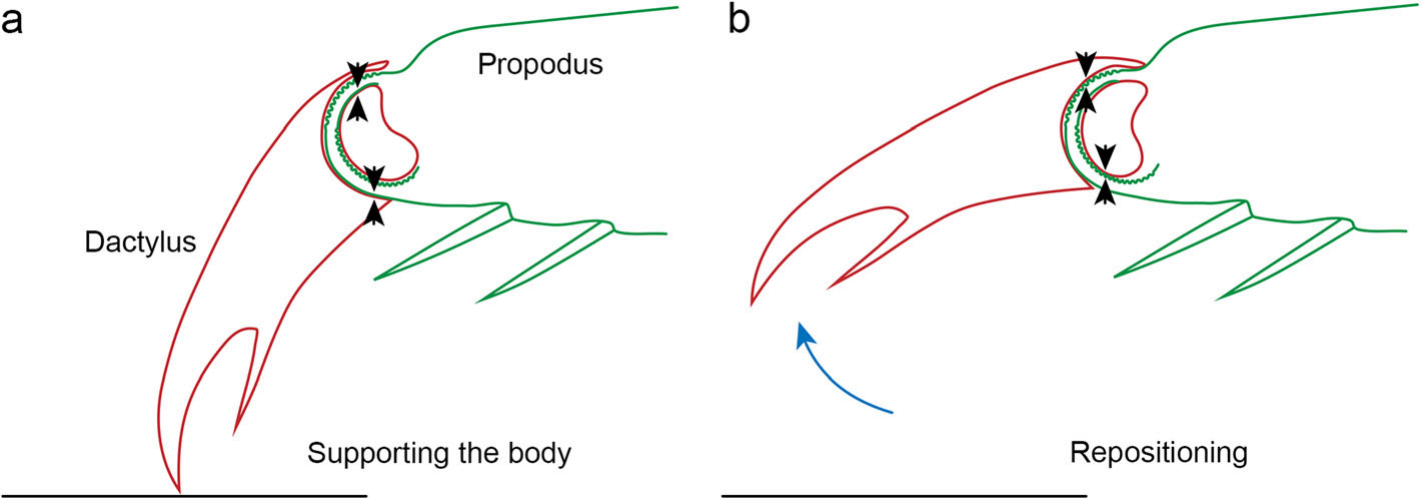
Predicted loading of contacting surfaces in the propodus-dactylus joint during locomotion. **a** During standing and the effective stroke of the pereopod, the process of the propodus pushes against the groove of the dactylus with its smooth surfaces: the convex ventral surface and the concave dorsal surface of its edge. **b** During the recovery stroke, as the pereopod is lifted off the substrate and repositioned, the propodus supports the dactylus with the patterned surfaces of the propodal process: its convex dorsal surface and its concave ventral surface. The dactylus can be repositioned during this stage as it responds to the roughness of the substrate (blue arrow)

During the effective stroke and while standing, we can expect the propodus to push against the dactylus as it supports the weight of the isopod. As the propodus is nearly parallel to the substrate in these cases, smooth surfaces of the propodal process lean against the groove of the dactylus. These are the concave surface of the dorsal part of the process and the convex surface of its ventral part (Fig. 8a). The patterned surfaces of the propodal process, on the other hand, are not expected to press against the groove.

During the recovery stroke, the propodus supports the dactylus, which is lifted from the substrate. In this case, we can expect the patterned surfaces of the propodal process to support the smooth groove of the dactylus (Fig. 8b). The dactylus may be repositioned during this stage.

Because the dactylus does not make large movements during the effective stroke, contacts between two smooth surfaces—the ventral segments of the propodal process and the groove on the dactylus—support the relatively large weight of the body without much sliding. During the recovery stroke, the much lighter dactylus is supported by contacts between the patterned surfaces of the propodus and the smooth surfaces of the dactylus, which slide as the dactylus moves.

### Surface patterning in other isopods

Joints were examined using SEM in three additional oniscidean species from different environments: *Cylisticus convexus*, *Ligidium hypnorum* and *Titanethes albus*. An aquatic sphaeromatidean (*S. serratum*) was also imaged.

**Fig. 9:**
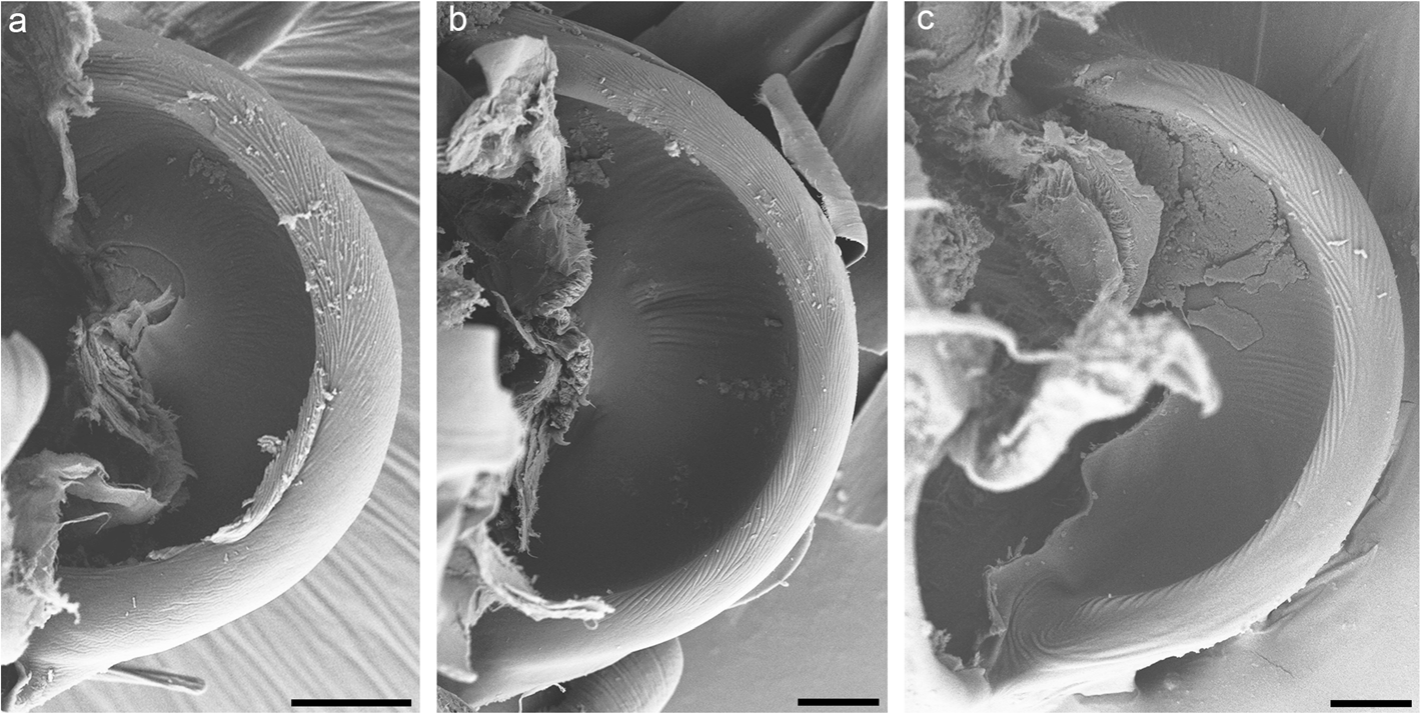
Propodal processes in other oniscideans. Scanning electron micrographs of the propodal processes in *Ligidium hypnorum* (**a**), *Titanethes albus* (**b**) and *Cylisticus convexus* (**c**). The processes are similar in shape and surface patterning, with slightly finer ridges in *T. albus*. Scale bars: 10 μm

Among the selected terrestrial isopods, *C. convexus* and *P. scaber* inhabit similar, mesic habitats and are completely terrestrial, *L. hypnorum* occurs in humid habitats, mostly near water, and *T. albus* is an amphibious cavernicolous species. In all the oniscideans examined, the propodus-dactylus joints resemble those of *P. scaber* in their structure and the patterning of the propodal processes (Fig. 9). The spatial frequencies of the ridges differ only slightly among species (summarized in Table 2).

**Table 2:** Sizes and ridge patterns of propodal processes in different isopod species

Species	Number of specimens/number of measurements per specimen	Suborder	Radius of the propodal process	Spatial frequency of ridges on the propodal process (mean ± standard deviation)
*Cylisticus convexus*	2/10	Oniscidea	35 μm	780 ± 115 nm
*Ligidium hypnorum*	2/10	Oniscidea	25 μm	400 ± 90 nm
*Porcellio scaber*	4/10	Oniscidea	40 μm	610 ± 150 nm
*Titanethes albus*	2/10	Oniscidea	40 μm	450 ± 90 nm
*Sphaeroma serratum*	1/15	Sphaeromatidea	130 μm	450 ± 50 nm

**Fig. 10:**
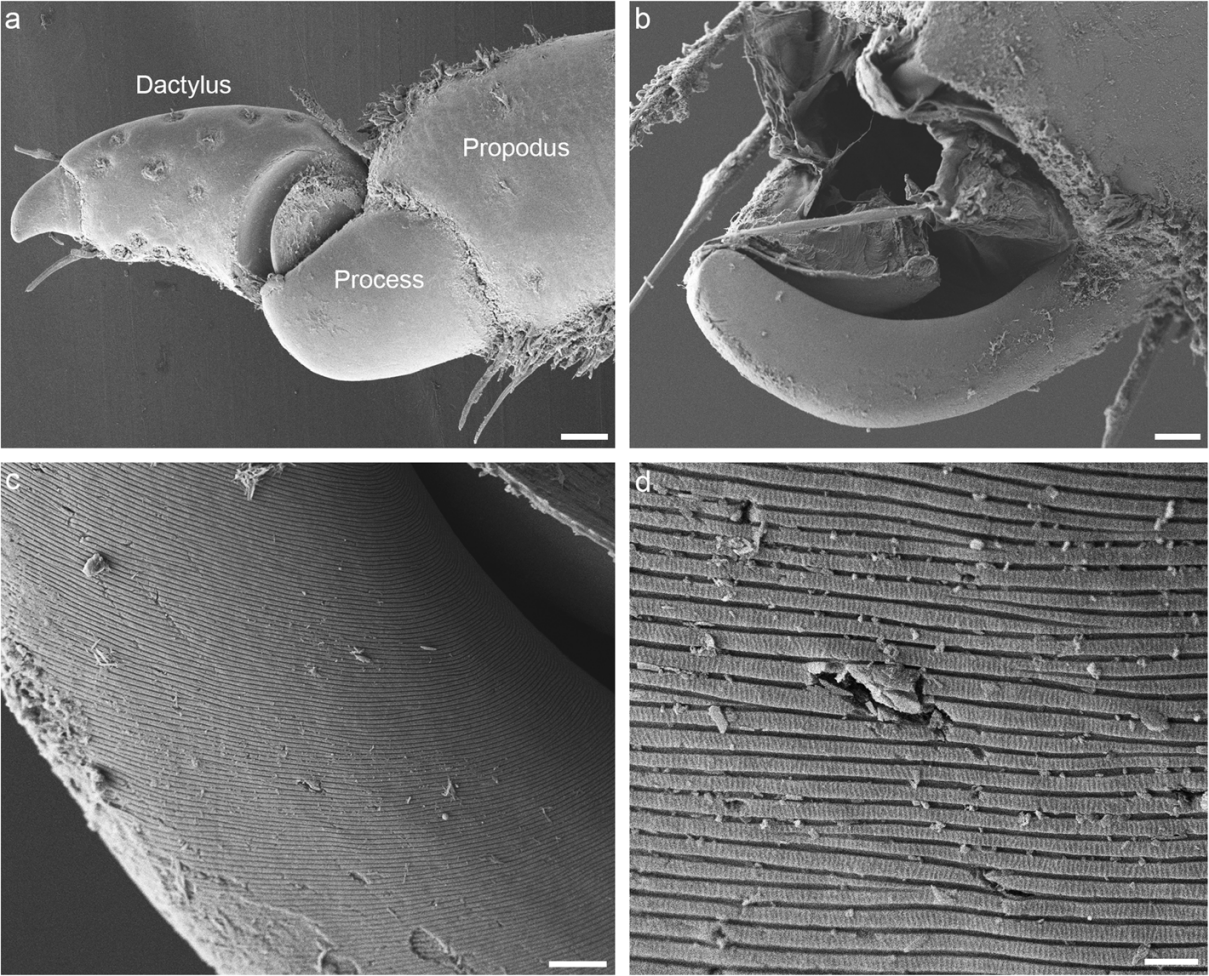
Propodus-dactylus joint in *Sphaeroma serratum.* **a** Scanning electron micrograph of the joint between the propodus and the dactylus in the aquatic sphaeromatid *S. serratum*. The dorsal side of the pereopod is at the top of the image. The propodal process does not form a full semicircle. Its thick edge fits into a smooth, semicircular groove on the dactylus. **b** Propodal process with the dactylus removed. Its curved ventral edge is thickened. **c** Higher magnification of the thick edge. Its surface is patterned and forms parallel ridges. **d** Ridges on the propodal process in *S. serratum* appear flat and cross-striated. Particles are trapped in the grooves between them. Scale bars: 50 μm (a), 20 μm (b), 5 μm (c), 1 μm (d)

In the marine isopod *S. serratum*, the propodal process is not fully semicircular. Instead, it extends only over the ventral half of the joint (Fig. 10). Despite this difference, the propodal process in *S. serratum* is patterned as in terrestrial isopods; its dorsal and medial surfaces are patterned with ridges of similar size and orientation as in oniscideans (Fig. 10), and its ventral surface is smooth.

## Discussion

The dactylus on the oniscidean pereopod slides along the edge of a process on the posterior portion of the propodus. This process and the corresponding groove on the dactylus are almost perfectly semicircular. The surface of the propodal process is textured with a pattern of minute ridges. The ridges are only present on surfaces that do not bear the bulk of the load, while the heavily loaded surfaces are smooth. This is the case in all the oniscideans studied, regardless of their habitats, as well as in the examined aquatic isopods.

It would be difficult to demonstrate unequivocally that the ridges have an adaptive function. Nevertheless, we can take the liberty of discussing their possible adaptive value. Their universal presence and distribution on sliding surfaces suggest that they facilitate the sliding of the dactylus along the propodal process. In this respect, the pattern of ridges can have two functions that are not mutually exclusive: it may reduce (1) wear or (2) friction.

Pairs of sliding surfaces, where one is smooth and the other is patterned with ridges or grooves, can reduce wear by removing particles that become trapped between them [14, 15]. In engineered nonlubricated bearings, grooves oriented at an angle to the direction of sliding eliminate small wear particles that accumulate in the grooves. As the particles are removed, they do not interact with the contacting surfaces, which would lead to increased wear and friction [15]. In bearings that operate underwater, such as the hydrodynamic bearings of ship propellers, grooves in the bushing—in addition to supplying water and providing cooling—also remove particles that enter the bearing. Restricting the grooves to the upper half of the bushing and keeping the bottom half smooth greatly increases the load-carrying capacity in such bearings [16]. This arrangement of grooves resembles the condition in isopods. The ridges in the joints of isopods may serve a similar function. This is plausible, as the same pattern is found not only in terrestrial species but also in amphibious and aquatic isopods.

The function of ridges in particle removal can be confirmed by observation. In SEM images, it can be seen that small particles are indeed lodged in the grooves between the ridges (Fig. 4). The grooves are so fine that they can accommodate only very small particles a few hundred nanometers in diameter. However, it makes sense for the grooves to be similar in size to the gap between the sliding surfaces, as larger particles will not lodge between them and therefore will not pose a problem.

Reducing friction is another possible function of the ridges. Measurements in artificial systems of similar dimensions have demonstrated that when surfaces textured with minute ridges slide against smooth probes, they have smaller coefficients of friction when compared to smooth surfaces [14, 17].

This effect can be attributed to the small real contact area between a textured and a smooth surface. On the microscopic scale, texturing that leads to a reduction in the real contact area reduces the intramolecular adhesion forces between dry contact surfaces, resulting in lower friction. In large systems supporting heavy loads, textured sliding surfaces may not be as beneficial, as rough surfaces can interlock and press into the contact surfaces, increasing friction and wear. Surface texturing is employed in such tribological systems—at least in machine elements—primarily for lubrication and the removal of wear particles [18, 19].

The propodus-dactylus joints in *P. scaber* are too small and complex to allow controlled friction measurements. However, we can look at insect joints for comparison. A somewhat similar surface texture has been described in the joint between the femur and tibia of orthopterans. In this case, parallel ridges approximately 2 μm thick with a distance of approximately 2 μm between each other were observed [20, 21]. A control joint in which both contact surfaces are smooth is naturally not available for measurement. Nevertheless, the study of polyurethane replicas of surfaces in the orthopteran joint showed that friction is reduced in contacts between a textured replica surface and a smooth surface compared to contacts between two smooth surfaces [20]. We can expect a similar effect in the joints of terrestrial isopods. The ridges may reduce the real contact area and thus reduce friction without increasing mechanical interlocking since one of the contact surfaces in the isopod joints is always smooth.

The movement of pereopods during walking in *P. scaber* greatly resembles descriptions in the amphibious *Ligia oceanica* and the marine *Janiralata occidentalis* and *Janiralata solasteri* [2, 12]. Although *P. scaber* was one of the isopod species studied by Hessler [2], he reported predominantly on *Janiralata*, as he concluded that limb morphologies and mechanisms of locomotion are similar in most isopods. This conclusion is largely confirmed by the present study. As the animal moves forward, individual pereopods are shifted as a consequence of rotation of the basis relative to the coxal plate [2]. The most anterior and the most posterior pereopods utilize extension and flexion of the ischium in propelling the body forward in addition to rotation of the basis. The position of the dactylus during walking has not previously been observed due to its small size and the limited resolution of high-speed recordings, even in species as large as *L. oceanica* [12]. As demonstrated in this work, the dactylus remains flexed throughout the effective stroke in *P. scaber*, as correctly assumed in previous studies [2, 12].

Because only the propodal surface is textured, the smooth areas of the propodal process press towards the substrate when the animal stands or walks, regardless of the angle between the dactylus and the propodus. This is likely the predominant way in which the joint is used, although woodlice also climb and cling to the substrate with their claws. Contact between two smooth surfaces in load-bearing areas of the joint may aid in force dissipation. As the real contact area between two smooth surfaces is greater, the force per unit of area is smaller. Since the propodus-dactylus joint in *P. scaber* does not move much when it is loaded—for example, when the animal is standing or during the effective stroke of walking—the greater friction or wear between two smooth surfaces may not be as relevant. Thus, the observed distribution of smooth and textured surfaces on the propodal arch in *P. scaber* is optimal for durability, enabling a large loading capacity while keeping the joint clean.

The study of patterned joint surfaces in terrestrial isopods could be a good starting point for further research that deepens our understanding of tribology at small scales and the evolutionary optimization of sliding surfaces for reduced friction and wear. The selective texturing of the joint between the propodus and the dactylus could be mimicked in bearings of small machine elements.

## Conclusions

The present study is the first description of the unique propodus-dactylus joint in terrestrial isopods at the microscopic level. The joint allows rotation of the dactylus in a single plane, powered by two antagonistic muscles. The edge of a semicircular process on the propodus fits into a groove on the dactylus, forming a guide along which the dactylus rotates.

The sliding surfaces on the propodal process are textured and form minute parallel ridges. This texturing is not uniform; while the surfaces that support the light dactylus during its repositioning are textured, the surfaces that support the weight of the body during standing and locomotion are smooth. The sliding surfaces of the dactylus, on the other hand, are entirely smooth. Possible contacts between sliding surfaces in the joint are therefore of two types: (1) between a textured surface and a smooth surface and (2) between two smooth surfaces. The first type is present in areas of the joint that are not heavily loaded during locomotion, and the second type is present in areas that bear heavy loads.

Two likely functions can be proposed for the ridges: (1) the reduction of wear by eliminating small particles and (2) the reduction of friction by reducing the real contact area between the sliding surfaces. The elimination of particles was confirmed by direct observation. The reduction of friction is supported by data obtained in similar biological and technical systems but eludes experimental demonstration in isopods at this time.

## Data Availability

The datasets used and/or analyzed during the current study are available from the corresponding author on reasonable request.
